# Olfactory expression of trace amine-associated receptors requires cooperative *cis*-acting enhancers

**DOI:** 10.1038/s41467-021-23824-3

**Published:** 2021-06-18

**Authors:** Ami Shah, Madison Ratkowski, Alessandro Rosa, Paul Feinstein, Thomas Bozza

**Affiliations:** 1 Department of Neurobiology, Northwestern University, Evanston, IL USA; 2 The Graduate Center Programs in Biochemistry, Biology and CUNY Neuroscience Collaborative, New York, NY USA; 3 Department of Biological Sciences, Hunter College, City University of New York, New York, NY USA; 4 Chemistry of Life Processes Institute, Northwestern University, Evanston, IL USA

**Keywords:** Gene regulation, Olfactory receptors

## Abstract

Olfactory sensory neurons express a large family of odorant receptors (ORs) and a small family of trace amine-associated receptors (TAARs). While both families are subject to so-called singular expression (expression of one allele of one gene), the mechanisms underlying TAAR gene choice remain obscure. Here, we report the identification of two conserved sequence elements in the mouse TAAR cluster (T-elements) that are required for TAAR gene expression. We observed that cell-type-specific expression of a TAAR-derived transgene required either T-element. Moreover, deleting either element reduced or abolished expression of a subset of TAAR genes, while deleting both elements abolished olfactory expression of all TAARs in *cis* with the mutation. The T-elements exhibit several features of known OR enhancers but also contain highly conserved, unique sequence motifs. Our data demonstrate that TAAR gene expression requires two cooperative *cis*-acting enhancers and suggest that ORs and TAARs share similar mechanisms of singular expression.

## Abstract (web summary)

How olfactory sensory neurons express one allele of one TAAR gene is not well understood. Here the authors identify two cooperative *cis*-acting enhancers that govern TAAR gene choice and that share both similarities and differences with known olfactory enhancers.

## Introduction

A vast majority of vertebrate genes are expressed from both the maternal and paternal alleles. However, a subset of genes is expressed from a single allele, a phenomenon referred to as monoallelic expression. For monoallelically expressed genes, the choice of expressed allele can be deterministic or random^1,2^. While monoallelic expression plays a central role in dynamic gene regulation, cell-type specification, phenotypic variability, and disease pathogenesis^3^, the mechanisms governing random monoallelic gene choice are ill-defined.

The mammalian olfactory system provides an intriguing example of random monoallelic expression. Odor detection is mediated by a large repertoire of olfactory receptor genes—over 1000 genes in mice. Each mouse olfactory sensory neuron (OSN) in the nasal cavity chooses to express one allele of one olfactory receptor gene from the repertoire of over 2000 alleles—so-called singular expression^4^. Olfactory receptors come in two phylogenetically distinct clades: odorant receptors (ORs) and Trace Amine-Associated Receptors (TAARs), both of which are subject to singular expression^5,6^.

The mechanisms of singular expression have been studied most extensively using mouse ORs as a model. The ORs come in two phylogenetically distinct families^7–10^. The class I OR family comprises roughly 130 intact genes located in a single large gene cluster, while the class II family comprises over 900 intact genes, most of which are located in over 50 clusters scattered throughout the genome^11^. The expression of some OR genes depends on short, proximal regulatory sequences^12,13^, while other genes require long-range, *cis*-acting enhancers^14^. The first identified enhancer, the H element, is located approximately 55 kilobases (kb) upstream of the MOR28 (*Olfr1507*) gene cluster^14,15^. Since the discovery of H, scores of similar enhancers have been identified scattered in and around OR gene clusters^16–21^. These include the P element in the P2 (*Olfr17*) cluster, the A/J-core element in the class I OR cluster^20,22^ and the large set of Greek Islands^19,21^.

These long-range enhancers and some proximal promoters contain conserved DNA sequence motifs, such as Olf1/Ebf (O/E)-like and homeodomain binding (HD) sites that have been implicated in olfactory-specific gene choice^17,23–26^. In addition, OR gene choice is partly dependent on the LIM homeodomain protein transcription factor Lhx2, which binds to the HD sites of OR promoters and enhancers^27–29^. Interestingly, the Greek Islands are enriched in co-bound O/E and HD sites^21^. The H (core), P, and A (J core) enhancers contain multiple copies of a characteristic HD site (TAATGA), including an extended HD sequence, CTTTTTAATGA^17,30^. Two of the HD sites in the H element are required for its enhancer activity^31^. Moreover, inserting multiple tandem copies of the HD site into an OR transgene dramatically increases probability of expression^17,30,32^. Thus, HD sites are a critical determinant for OR choice.

Beyond these sequence motifs, epigenetic mechanisms have been proposed to play a central role in OR gene regulation. The available evidence suggests that singular expression involves a complex interaction between enhancers and proximal promoters to selectively release a single OR allele from epigenetic repression^19,21,33–35^. OR gene clusters are marked as constitutive heterochromatin by specific histone modifications (H4K20me3 and H3K9me3) which silence gene transcription^33^. This repression correlates with a change in nuclear localization of OR loci from the nuclear lamina to internal nuclear aggregates^36^. Conversely, expression of a given OR locus is correlated with a change from repressive to permissive H3K4me3 histone marks, indicating a change in histone state correlated with singular expression^33^. Thus, epigenetic repression and specific nuclear localization may be central to the mechanism of OR gene choice.

By contrast, little is known about the mechanism of TAAR gene choice. The TAAR family in mouse comprises 15 genes, all of which are located in a single genomic cluster with no intervening genes^37^. Like ORs, all of the TAARs (except *Taar1*) are subject to singular expression in subsets of OSNs^6,37–39^. Unlike ORs, TAARs exhibit some differences that are not easily reconciled with features of OR gene choice. First, the TAAR gene cluster lacks the enrichment in epigenetic marks that are thought to silence class II OR gene clusters, at least when looking across all mature OSNs^40^. Second, it has been suggested that TAAR-deletion alleles behave differently from OR-deletion alleles—that OSNs expressing an OR deletion allele switch to express an alternate gene and silence the initially chosen locus, while TAAR deletion alleles appear to remain active^40^. Third, while most class I/II OR genes are located in several internal heterochromatic centers in the nucleus, the TAAR genes are located preferentially at the nuclear lamina prior to choice^36,41^. In fact, it has been suggested that the mechanism of TAAR gene expression is fundamentally different from that of ORs^40^. However, the genetic mechanisms underlying TAAR gene regulation are poorly characterized, and no enhancer or promoter sequences have been identified that are necessary to promote TAAR gene expression.

Here, we identify two phylogenetically conserved enhancers in the TAAR gene cluster and show that they are necessary and sufficient to promote TAAR gene expression. The TAAR enhancers exhibit both unique and shared features with known OR enhancers. Our findings establish a powerful model for studying olfactory receptor gene choice and suggest that TAAR and OR gene expression take place via a similar basic mechanism.

## Results

### TAAR4 transgene expression requires an enhancer

**Fig. 1:**
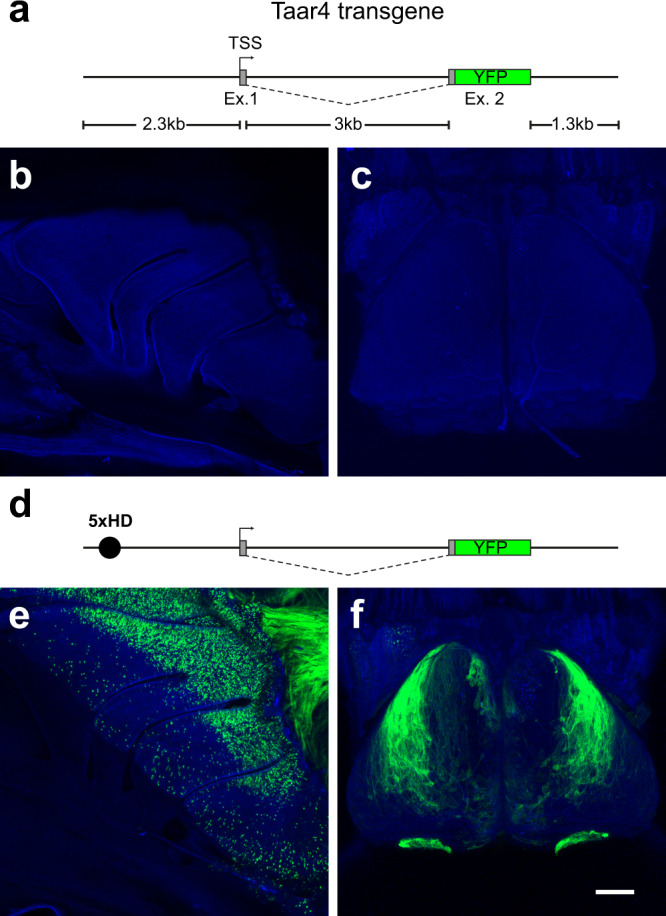
TAAR4 transgene expression requires a strong OR enhancer. A transgene derived from the endogenous Taar4 locus is not sufficient to drive reporter expression. **a** Schematic of the transgene backbone in which the Taar4 coding sequence in exon 2 (Ex. 2) is replaced with that of YFP (green box). Non-coding regions shown in gray. Defined transcription start site (TSS) is indicated (arrow). **b** Confocal image showing a wholemount of the olfactory turbinates of a ΔT4-YFPtg mouse showing lack of labeled OSNs. **c** Confocal image showing a wholemount of the dorsal olfactory bulb showing no labeled axons. **d** Diagram of the 5xHD-ΔT4-YFPtg, in which a strong OR enhancer (5xHD; black circle) is placed upstream of the putative promoter. **e** Wholemount fluorescence image of the olfactory turbinates showing robust expression of YFP in OSNs primarily in the dorsal olfactory epithelium. **f** YFP+ axons of OSNs primarily innervate the dorsolateral olfactory bulb and accessory olfactory bulb. Scale bar in **f** = 500 µm in **b**, **c**, **e**, and **f**.

To begin defining the minimal promoter sequences required for TAAR gene expression, we generated a TAAR4 transgene consisting of a genomic fragment encompassing ~2.3 kb upstream of the *Taar4* transcription start site (determined by 5′ RACE), the single intron, and the endogenous polyadenylation site (Fig. 1a). The transgene was modified so that the Taar4 coding sequence was deleted and replaced with that of YFP. The resulting transgene (ΔT4-YFPtg) failed to be expressed in 5 independent founder lines (Fig. 1b, c). This suggested that the included putative promoter was insufficient to impart gene choice, or that the transgene was otherwise non-functional.

To test the functionality of the transgene, we appended a strong OR enhancer (5x repeat of an extended homeodomain binding site), 5×21 HD^30^, to the 5′ end of the ΔT4-YFPtg (Fig. 1d). The resulting transgene (5xHD-ΔT4-YFPtg) was robustly expressed in OSNs of the olfactory epithelium in 5 out of 5 independent lines (Fig. 1e). All but one of the transgenic lines showed a characteristic pattern of expression, with robust labeling of OSNs of the dorsal main olfactory epithelium and in VSNs of the vomeronasal organ (Fig. 1e; Fig. S1). They also shared a common pattern of glomerular projections to the dorsolateral olfactory bulb and accessory olfactory bulb (Fig. 1f and Fig. S1). This indicated that the ΔT4-YFPtg transgene is functional for gene expression when an olfactory enhancer sequence is provided.

### Identification of putative TAAR enhancers

**Fig. 2:**
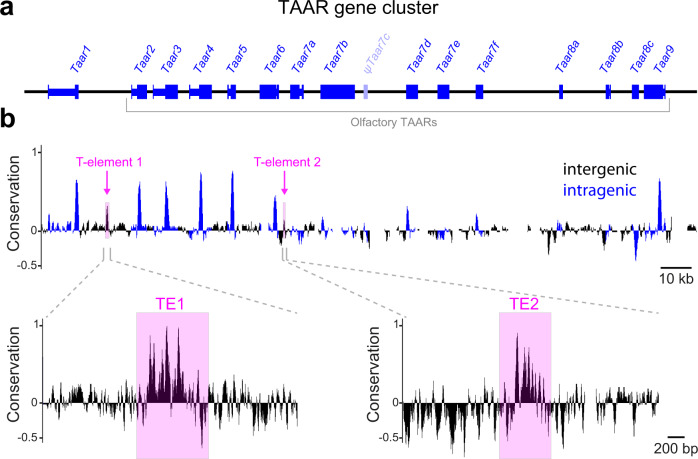
Identification of putative enhancers in the TAAR cluster. **a** Gene structure of all 15 intact TAARs (blue) and the pseudogene ψ*Taar7c* (light blue). Exons (thick lines) and introns (thin lines) are indicated. **b** Basewise conservation across eutherian mammals in the TAAR cluster determined using PhyloP. Regions within known genes are shown in blue, intergenic regions in black. Two intergenic regions that have high conservation peaks and HD sequences (TE1 and TE2) are highlighted (magenta). Conservation track is expanded below.

By analogy to canonical ORs, we reasoned that TAAR gene expression may require *cis*-acting enhancers that are located in the cluster but that are missing from the TAAR4 transgene. To identify putative enhancers, we searched the mouse TAAR cluster between the conserved genes *Vnn1* and *Stx7* for sequences that were: (1) conserved across eutherian mammals, (2) non-repetitive, and (3) intergenic (i.e., outside known coding sequences, UTRs, or pseudogene fragments; Fig. 2a). To search for conservation, we used a combination of basewise sequence comparison (percent identity plots; PipMaker^42^ between mouse and human, and the phyloP conservation track for 40 eutherian mammals in the UCSC database) (Fig. 2b). We identified two conserved intergenic regions that matched our criteria—one between *Taar1* and *Taar2* and one between *Taar6* and *Taar7a* (Fig. 2b). These sequences were named T-elements 1 and 2 (TE1 and TE2), respectively.

### The T-elements share some features with OR enhancers

**Fig. 3:**
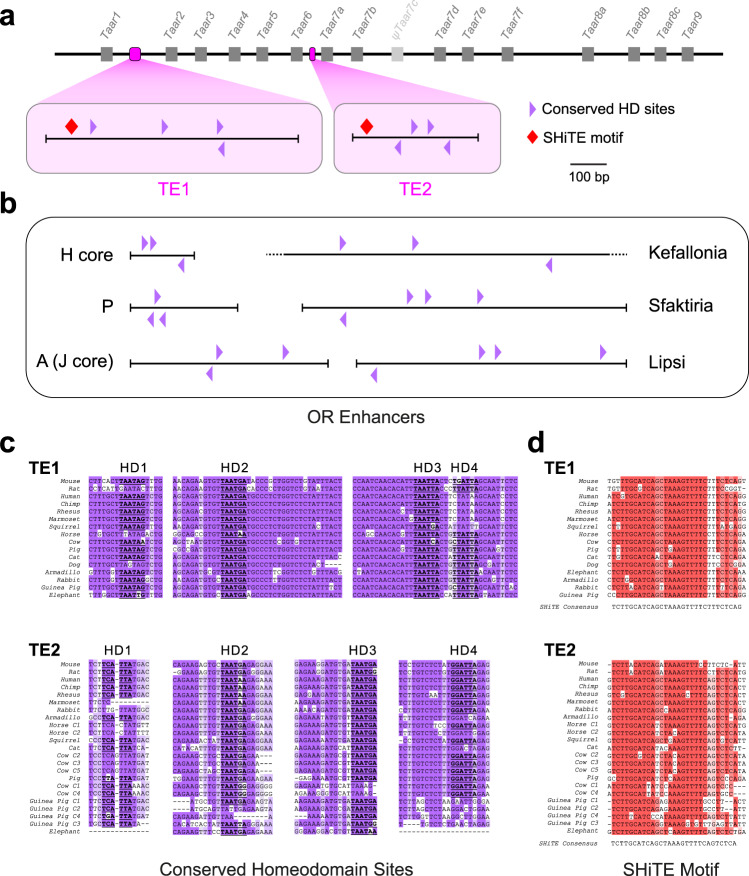
The T-elements comprise conserved homeodomain binding sites and a novel shared motif. **a** Schematic of the TAAR cluster showing relative positions of TE1 and TE2 (magenta). Genes are indicated by gray boxes. Schematic of the T-elements (bottom) showing placement of phylogenetically conserved HD sites (purple triangles). The T-elements also contain a highly conserved, shared homology, the SHiTE motif (red diamonds). **b** Schematic of known functional OR enhancers showing positions of conserved HD sites. **c** Alignments of segments of TE1 and TE2 from 16 mammalian species showing all conserved HD sites, HD1-4 in each. **d** Alignment of the TE1 and TE2 shared homology (SHiTE) motifs across representative mammalian species. Single alignment is split, top (TE1) and bottom (TE2).

Known OR enhancers contain HD and O/E-like binding sites^17,20,27^. We therefore searched for putative HD and O/E-like sites in TE1 and TE2 and looked for conservation that might suggest function. Each element contains seven instances of the core HD binding motif TAATNN. In TE1, four of these copies are conserved including one instance each of TAATAG, TAATGA, TAATTA, and TAATCA (Fig. 3a, c and S2). Similarly, four HDs are conserved in TE2, one instance of TAATCC and a triad of TAATGA sequences (Fig. 3a, c and S2). This triple repeat of TAATGA located within 206 bp of each other (a motif that is known to influence choice^17,30^) is only found once in the TAAR cluster (in TE2). Similarly, there are conserved HDs in 6 known functional OR enhancers: H-core, P, A (J core) and the Greek Islands Lipsi, Sfaktiria and Kefallonia (Fig. 3b, c and S3). Thus, as with known OR enhancers, the T-elements contain multiple copies of conserved HD binding sites.

Next, we searched TE1 and TE2 for O/E-like sites that closely match the degenerate consensus sequence YCCCNNGGGR^21,43–45^. We identified three such sites in both mouse TE1 and TE2; however, they were not conserved across species. In contrast, we could easily locate well-conserved O/E-like sites in all of the OR enhancers except H (Fig. S4), which contains an O/E-like site only in mouse. Thus, unlike OR enhancers, TE1 and TE2 appear to lack recognizable, conserved O/E-like sites.

**Fig. 4:**
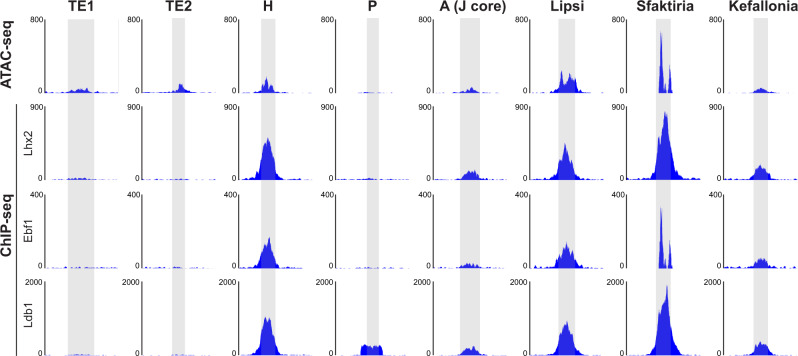
TE1 and TE2 share some but not all epigenetic features of OR enhancers. ATAC-seq and ChIP-seq data from all mature OSNs^21,27^ in genomic regions corresponding to TE1 and TE2, and known functional OR enhancers: H, P, A (J core), Lipsi, Sfaktiria and Kefallonia. T-elements show similar chromatin accessibility, but lack Lhx2, Ebf1 (O/E), and Ldb1 binding that is observed in most of the known functional OR enhancers.

The Greek Islands are defined in part by chromatin accessibility peaks and binding of transcription factors Lhx2, Ldb1, and Ebf1^21^. To examine whether the T-elements share such features with the 6 known functional OR enhancers, we analyzed published ATAC-seq and ChIP-seq data from mature (OMP-expressing) OSNs^21,27^. In this data set, both TE1 and TE2 show similar accessibility to Kefallonia, A (J core) and H (Fig. 4). However, neither TE1 nor TE2 was as enriched for binding of Lhx2, Ebf1, or Ldb1 as most of the known OR enhancers. This lack of binding was also observed for the P element. Thus, averaged across all OMP-expressing OSNs, TE1 and TE2 exhibit some, but not all, features of known OR enhancers. It should be noted that TAAR OSNs represent a small fraction of the total OSN pool and some of these enrichments of binding may only be observed in TAAR-expressing OSNs (see Discussion).

### The T-elements contain a unique, shared motif

Our analysis also revealed a ~30 bp block of conserved sequence that is common between the two T-elements (Fig. 3a, d). This motif, referred to as Shared Homology in the T-Elements (SHiTE), contains two tandem conserved sequences, TTGCATCA and TAAAGTTTTC. We searched for the SHiTE motif against a database of known OR enhancers including the 63 Greek Islands using FIMO (Meme suite^46^) and BLAST. No significant matches were found (FDR *q* < 0.05). The only close match (*q* = 0.086) was in the Greek Island Evia and consisted of a stretch of homology encompassing the SHiTE motif, AAAGTTTTCT. In a genome wide search, we found no matches to the full-length SHiTE motif outside of the TAAR cluster. While matches to shorter stretches of SHiTE (often including AAAGTTT) are found throughout the genome, the full SHiTE motif seems to be unique to the TAAR elements.

### The T-elements drive transgene expression

**Fig. 5:**
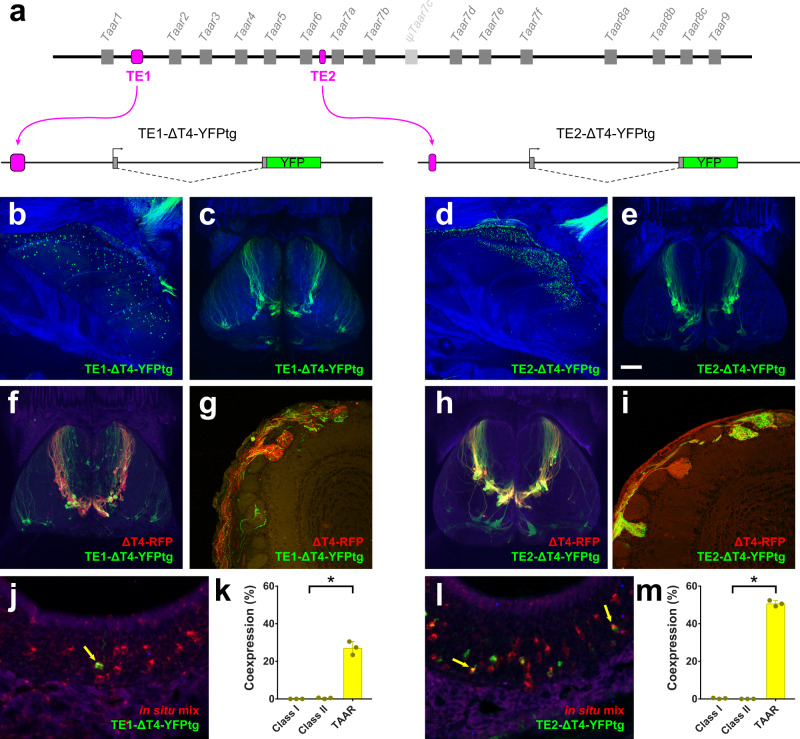
TE1 and TE2 drive expression of the TAAR4 transgene. **a** Top, schematic of TAAR cluster showing placement of TE1 or TE2 (magenta). Bottom, diagram of ΔT4-YFP transgenes in which TE1 and TE2 are inserted upstream of the Taar4 promoter and YFP coding sequence (green). **b**–**e** Wholemount confocal images of olfactory epithelium and olfactory bulb of TE1-ΔT4-YFPtg and TE2-ΔT4-YFPtg mice showing YFP expression in OSNs in the epithelium and labeled axons and glomeruli in the dorsal olfactory bulb. OSN axons project to a cluster of dorsal glomeruli, reminiscent of the endogenous TAAR glomeruli. **f** Confocal image of dorsal olfactory bulbs, and **g** histological section, of a mouse that has the TE1-ΔT4-YFPtg and a ΔT4-RFP targeted allele. RFP+ and YFP+ axons target the same glomeruli. **h** Confocal image of dorsal olfactory bulbs, and **i** histological section, of a mouse that has the TE2-ΔT4-YFPtg and a ΔT4-RFP targeted allele. RFP+ and YFP+ axons target the same glomeruli. **j**, **l** Combined in situ hybridization (TAAR probe mix, red) and immunohistochemistry for YFP (green) in histological sections of olfactory epithelium from TE1-ΔT4-YFPtg (**j**) and TE2-ΔT4-YFPtg (**l**) mice. Co-expressing cells indicated by yellow arrows. **k**, **m** Fraction of YFP+ cells that label with the class I, class II, or TAAR probe pools in TE1-ΔT4-YFPtg (**k**) and TE2-ΔT4-YFPtg (**m**) animals (*n* = 3 mice). Error bars indicate mean ± SEM. *p < 0.0001; two-sided, one-way ANOVA, Dunn–Šidák correction for multiple comparisons. Scale bar in **e** = 500 µm in **b**, **c**, **d**, **e,** **f,** and **h**; 100 µm in **g** and **i**; 25 µm in **j** and **l**.

To test whether the T-elements function as enhancers for TAAR genes, we appended TE1 (698 bp) or TE2 (345 bp) to the 5′ end of the ΔT4-YFPtg to see if each element could rescue transgene expression (Fig. 5a). Interestingly, addition of either TE1 or TE2 drove robust transgene expression in OSNs (Fig. 5b, d). TE1 (TE1-ΔT4-YFPtg) drove strong expression in 2 of 3 independent transgenic lines, with sparsely labeled neurons located throughout the olfactory epithelium (Fig. 5b). TE2 (TE2-ΔT4-YFPtg) drove strong expression in 4 of 5 lines, with one line showing the highest numbers of OSNs in the dorsal zone of the epithelium (Fig. 5d), and others showing significant expression in more ventral regions, and in the septal organ (Fig. S1). (Because TAAR expression has not been reported in the septal organ, this likely represents ectopic expression.) Thus, like the 5×21 HD enhancer, both TE1 and TE2 can rescue the expression of the ΔT4-YFPtg, indicating that they have enhancer activity.

### The T-elements drive expression in TAAR sensory neurons

Because the *Taar4*-derived transgene lacks a receptor coding sequence, YFP expressing OSNs would be expected to express an alternate receptor gene, which would then direct axons to specific glomeruli in the olfactory bulb^14,47,48^. Consequently, the pattern of innervation in the bulb provides information about which cell types express the transgene^6,16,49^.

We noted that, in specific T-element transgenic lines, YFP-labeled axons converged to multiple glomeruli in the dorsomedial olfactory bulb (Fig. 5c, e) in a pattern that resembled the distribution of dorsal TAAR glomeruli^6,40,41^. This pattern of innervation was not seen in any of the 5xHD-ΔT4-YFPtg lines, which tend to label glomeruli in the dorsal–lateral bulb (Fig. 1f) in a DII (dorsal class II) pattern^16^. To determine if T-element transgene-expressing OSNs were preferentially innervating TAAR glomeruli, we crossed one line for each transgene to the gene-targeted mouse strain, ΔT4-RFP, in which the coding sequence for TAAR4 is replaced with that of a red fluorescent marker, thereby labeling all dorsal TAAR glomeruli^6^. Wholemount and histological analysis showed that all RFP-labeled glomeruli were targeted by YFP-labeled axons (Fig. 5f–i). While the relative contribution of ΔT4 and transgenic axons (i.e., the fraction of red to green axons) varied across glomeruli, the preferential innervation of TAAR glomeruli indicated that transgene-expressing OSNs preferentially express TAARs.

To directly test for selective TAAR expression in transgene-labeled OSNs, we performed combined immunohistochemistry for YFP protein and in situ hybridization using pooled probes for 6 representative class I ORs, class II ORs, and TAARs (Fig. 5j, l). If expression of intact receptor genes were random across all receptor types, each probe pool should exhibit a similar, low level of co-expression. Contrary to this prediction, the co-expression rate observed with the TAAR-probe pool was significantly higher than that for the class I and class II pools. This was observed for both transgenes (Fig. 5k, m). Thus, for the lines analyzed, transgene-expressing OSNs exhibited a bias towards expressing TAAR genes. Taken together, the data indicate that the T-elements may influence the cell-type-specific expression of TAAR genes.

### The T-elements are required for TAAR gene expression

**Fig. 6:**
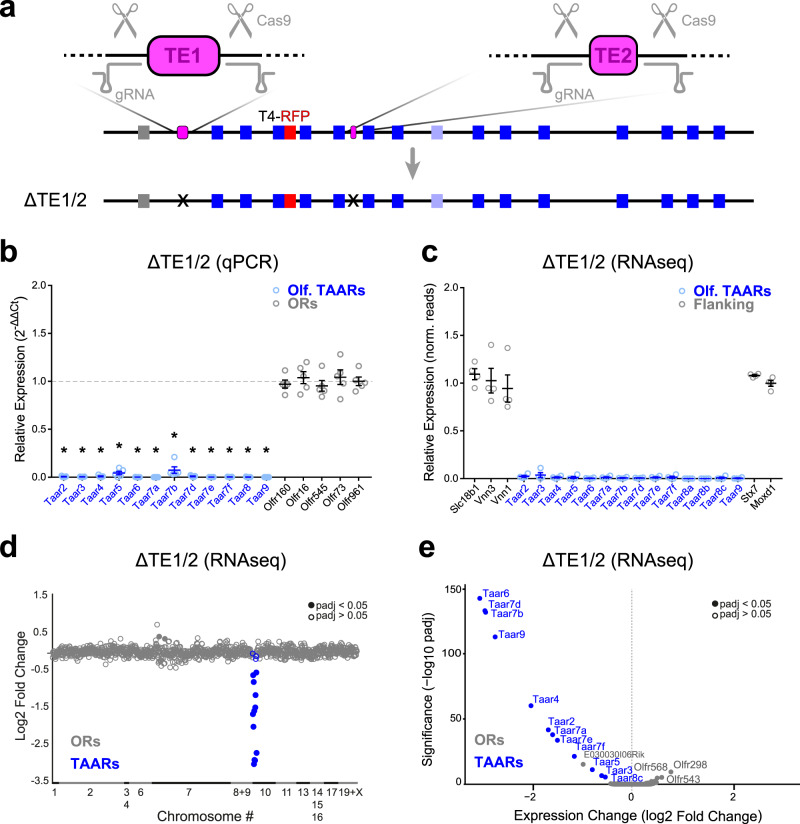
TE1 and TE2 are required for TAAR gene expression. CRISPR-based gene editing was used to generate a double, *cis* deletion of both TE1 and TE2. **a** Diagram showing locations of gRNAs (gray) designed to target PAM sites flanking TE1 and TE2 (magenta). The double deletion (ΔTE1/2) was generated on a chromosome harboring a previously targeted Taar4-RFP allele (red). Bottom, diagram of ΔTE1/2 in *cis* with Taar4-RFP. Deletion is marked (X). **b** Relative fold expression of TAAR (blue) and control OR (gray) genes measured using qPCR (2^-ΔΔCt^ method) from olfactory mucosa. Error bars indicate mean ± SEM. Taar8 primers amplify all 3 family members. *n* = 5 mice per genotype. *p < 0.0001; two-sided, one-way ANOVA, Dunn–Šidák correction for multiple comparisons. **c** Relative expression (Δ/wt_avg_ normalized counts from DESeq2) for olfactory TAAR genes (blue) and genes flanking the TAAR cluster (gray). Error bars indicate mean ± SEM. *n* = 4 mice per genotype. **d** Change in expression (log_2_ fold change) of all ORs (gray) and TAARs (blue) measured via RNAseq (*n* = 4 mice for each genotype). **e** Volcano plot (−log_10_ adjusted *p* value vs. log_2_ fold change) from DESeq2 analysis of RNAseq data from homozygous ΔTE1/2 and wild-type littermate control olfactory mucosa showing that TAAR genes are selectively affected by deletion. All genes with normalized counts >10 are plotted. Filled circles indicate genes with adjusted *p* values <0.05 two-sided (DESeq2) Wald test corrected for multiple comparisons (Benjamini and Hochberg). *p*-values for RNAseq data can be found in Source Data File.

To test whether TE1 and TE2 are necessary for TAAR gene expression, we generated deletions of each element alone, or together in *cis*, using CRISPR/Cas9-mediated gene editing. Guide RNAs were designed to PAM sequences flanking each of the elements (Fig. 6a). Gene editing was carried out in mice harboring modified alleles of *Taar1* (Taar1-YFP) and *Taar4* (Taar4-RFP), allowing us to track the expression of these genes in *cis* with the mutations. We isolated three mutations: (1) a double *cis* deletion of TE1 and TE2 (ΔTE1/2) linked to Taar4-RFP (Fig. 6a); (2) a deletion of TE1 (ΔTE1) linked to Taar1-YFP; and (3) a deletion of TE2 (ΔTE2) linked to Taar1-YFP (see below).

**Fig. 7:**
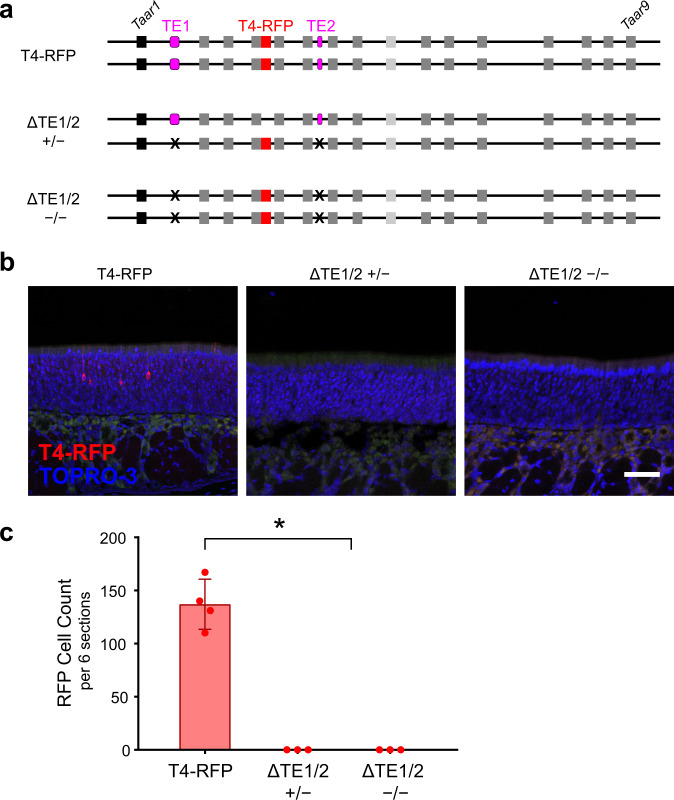
TE1 and TE2 influence TAAR gene expression in *cis* not *trans*. **a** Three genotypes used to test for *trans* effects of TAAR elements. TE1 and TE2 are shown (magenta). Deletions are indicated (X). Taar4-RFP targeted allele (red) is linked to the double deletion, ΔTE1/2. **b** Confocal images of representative olfactory epithelium sections showing RFP-labeled OSNs (red) in Taar4-RFP mice (left). Nuclei are labeled with TOPRO-3 (blue). No labeled OSNs are seen in homozygous (right) or heterozygous (middle) ΔTE1/2 mice (*n* = 3 mice per genotype). **c** Average number of RFP+ OSNs counted in 6 representative olfactory epithelium sections from Taar4-RFP (*n* = 4), ΔTE1/2 heterozygous mice (*n* = 3), and ΔTE1/2 homozygous mice (*n* = 3 mice). *p < 0.0001 two-sided, ANOVA with contrasts. Error bars indicate mean ± SEM. Scale bar = 50 µm.

We first analyzed ΔTE1/2 mice, in which both elements were deleted on the same chromosome in *cis* with the Taar4-RFP allele (Fig. 6a). Taar4-RFP (with intact elements) normally produces robust labeling of OSNs in the dorsal epithelium^6^ (Fig. 7). Notably, in ΔTE1/2 homozygous mice, no RFP expression was seen from the linked Taar4-RFP allele (Fig. 7). To define the extent of the effect of the double deletion, we measured expression of all TAARs in the olfactory mucosa of homozygous ΔTE1/2 mice via qPCR. In contrast to wild-type littermates, homozygous ΔTE1/2 mice showed no expression of TAAR genes, while expression of a set of control OR genes was unaffected (Fig. 6b, S5). This indicates that TE1 and/or TE2 are necessary for expression of TAARs in the olfactory epithelium.

To determine whether the effects of deleting the elements was TAAR-specific, we performed RNA-seq on olfactory mucosa from homozygous ΔTE1/2 and wild-type littermates. The element deletion did not affect expression of genes that immediately flank the TAAR cluster and that are expressed in the olfactory mucosa—*Slc18b1*, *Vnn1* and *Vnn3*, *Stx7* and *Moxd1* (Fig. 6c). In addition, the only olfactory receptors that exhibited significantly reduced expression in ΔTE1/2 mice were TAARs (Fig. 6d). Across all genes expressed in the olfactory mucosa, only one non-TAAR gene (*E030030I06Rik*) was significantly downregulated (Fig. 6e). Therefore, reduced expression caused by the ΔTE1/2 mutation is specific to the TAAR gene cluster.

To determine if TE1 and TE2 act exclusively in *cis* (on the same allele), we looked for RFP expression in heterozygous ΔTE1/2 mice in which the elements are present in *trans*, but lacking in *cis*, of the Taar4-RFP allele (Fig. 7a). We observed no RFP expression from the Taar4-RFP allele in heterozygous ΔTE1/2 mice (Fig. 7b, c), indicating that the elements function solely in *cis*.

Thus, knocking out both TE1 and TE2 in *cis* completely and selectively abolishes expression of the linked TAAR genes in the olfactory epithelium.

### The T-elements have a combinatorial effect on TAAR gene expression

**Fig. 8:**
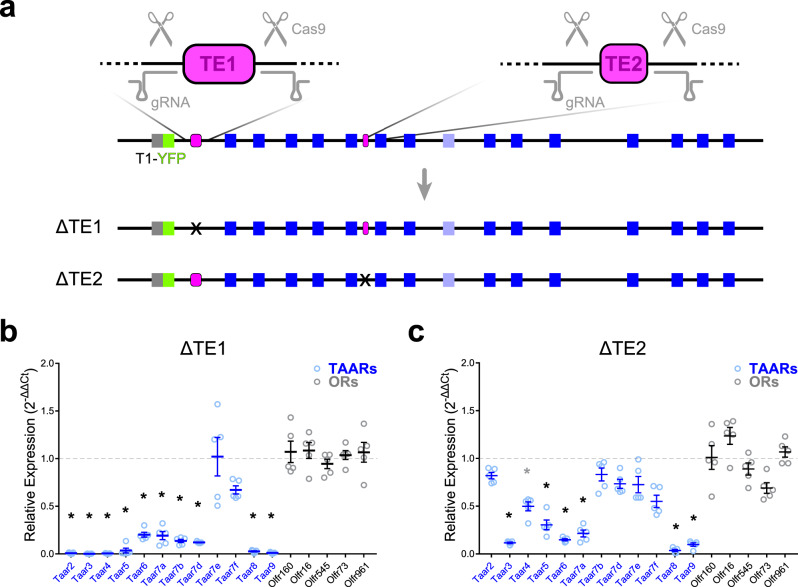
TAAR enhancers function cooperatively. **a** Generation of single T-element deletions via CRISPR. Locations of gRNAs (gray) are shown relative to TE1 and TE2 (magenta). The individual ΔTE1 and ΔTE2 mutations were generated on a chromosome harboring a previously targeted *Taar1-YFP* allele (green). Bottom, diagram of ΔTE1 and ΔTE2 in *cis* with Taar1-YFP. Deletion is marked (X). **b**, **c** Relative fold expression of TAAR (blue) and control OR (gray) genes measured using qPCR (2^-ΔΔCt^ method) from olfactory mucosa from ΔTE1 and ΔTE2 mice. Error bars indicate mean ± SEM. *Taar8* primers amplify all 3 family members. *n* = 5 mice per genotype. *p < 0.01; one-way ANOVA, Dunn–Šidák correction for multiple comparisons.

To define the specific contribution of TE1 and TE2 to TAAR gene expression, we quantified gene expression in the olfactory mucosa of single element deletion mice. Both the ΔTE1 and ΔTE2 mutations were generated independently in *cis* with a Taar1-YFP allele (Fig. 8a), allowing us to observe whether deleting either element induced *Taar1* gene expression in the epithelium (i.e., normally insulate *Taar1* from being chosen). However, we did not observe Taar1-YFP expression in the olfactory epithelium of either strain (not shown).

Next, we quantified olfactory TAAR expression via qPCR. ΔTE1 mice showed dramatically reduced expression of all TAAR genes except *Taar7e* and *Taar7f*, with the affected TAARs being essentially undetectable (Fig. 8b, S5). It is interesting to note that the effect of this deletion was not restricted to adjacent TAARs (e.g., *Taar2* and *Taar3*), but extended to the opposite end of the cluster (e.g., *Taar9*). Thus, TE1 has a strong influence on expression of almost all olfactory TAARs. On the other hand, ΔTE2 mice showed a significant reduction in expression of a different constellation of TAAR genes including *Taar3*, *4*, *5*, *6*, *7a*, *8a/b/c*, and *9* (Fig. 8c, S5). Several TAAR genes (*Taar2*, *7b*, *7d*, *7e*, *and 7f)* were relatively spared. Overall, the effect of the TE2 deletion was less pronounced than for TE1. The effects of the two deletions were distinct but overlapping—some genes (e.g., *Taar3*) were severely downregulated by both mutations, while *Taar7e* was generally spared by both. Interestingly, there was no clear relationship between the zone of expression and dependence on either element. For example, deletion of TE1 reduced expression of both dorsal (e.g., *Taar2* and *Taar9*) and ventral (e.g., *Taar7a* and *Taar7b*) genes. Thus, each element has differential and overlapping effects on TAAR gene expression.

Taken together, our data indicate that TE1 and TE2 are *cis*-acting enhancers that function in a combinatorial fashion to selectively promote olfactory expression of genes in the TAAR cluster.

## Discussion

Most of what is known about the mechanisms of olfactory singular expression has come from studying ORs. Consequently, the relationship between mechanisms of TAAR and OR gene choice is not well understood. Here, we identify two conserved enhancers in the TAAR gene cluster (TE1 and TE2) and show that deletion of both abolishes TAAR gene expression in OSNs. We further show that the enhancers function cooperatively, with each affecting an overlapping subset of TAAR genes. A recent study has identified similar regulatory sequences and corroborates these findings^50^. Interestingly, the T-elements share features with known OR enhancers (i.e., the presence of conserved HD binding motifs), but also contain conserved, common motifs that appear specific to the T-elements and that may underlie critical aspects of their function. Overall, our data indicate that TAAR and OR gene regulation share a common basic mechanism. Furthermore, the TAAR cluster is the only olfactory receptor gene cluster for which all the genetic elements that are required for expression are known. Therefore, the TAAR cluster provides a powerful model to explore mechanisms of olfactory receptor gene choice.

Previous studies have deleted single OR enhancers in a given gene cluster, and have shown distance-dependent, partial suppression of OR gene expression^15,18–20,22^. Deleting the enhancers H, P, and Lipsi in class II OR clusters impacts genes over a distance of ~200 kb^15,18,19^, while deleting A (J core) in the class I OR cluster affects genes as far away as 3 Mb^20,22^. In all cases, expression persists for many genes in the clusters. This has led to the idea that multiple enhancers are required to drive expression of all genes in a given cluster. While likely, this has not been shown directly and it is difficult to exclude the possibility that some genes in the cluster are simply independent of long-range enhancers. The effects of TE1 and TE2 provide an example of cooperative enhancer function that is required for expression of all genes in an olfactory cluster.

We show that, while the T-elements function together to regulate all olfactory TAAR genes, each element alone influences expression of distinct, overlapping sets of genes. The effect of single T-element deletions was not obviously correlated with distance from the enhancers—something that was also seen with deletion of the P element^18^. We note that the TAAR cluster is relatively small (~200 kb) compared with typical OR gene clusters (mean size ~1.2 Mb^7^), which means that TE1 and TE2 could affect the entire TAAR cluster even if their range is similar to that of the OR enhancers.

Our previous data show that TAAR genes residing on the distal ends of the cluster are expressed in the dorsal zone of the olfactory epithelium, while those in the middle of the cluster (near TE2) are expressed ventrally or broadly across zones^6^. This might indicate that TE2 influences zonal expression and/or selectively drives expression of the interior genes. Interestingly, we did not observe a clear relationship between zone of expression and susceptibility to deletion of TE1 or TE2—both single element deletions affected dorsally and ventrally expressed TAARs. Additionally, our TE2-containing TAAR4 transgene reliably drove expression in the dorsal epithelium, while the TE1 transgene tended to drive expression more broadly. Thus, the transgene expression patterns do not support a simple model in which TE2 is the sole director of ventral/broad expression, as suggested by its position among the ventrally/broadly expressed genes. Future studies examining promoter/enhancer interactions may shed light on what determines zonal expression of TAAR genes.

Most putative OR enhancers (i.e., the Greek Islands) have been defined operationally based on accessibility and transcription factor binding^19,21^. A handful of enhancers have been functionally defined by adding the sequences to transgenes or by deleting the sequences in mice^15,17–20,22^. It is worth noting that the lack of complete silencing of OR gene expression in all previous enhancer deletions leaves open questions about the actual minimal sequences that are required for enhancer function. Transgenic expression reports how a specific enhancer fragment can function to promote expression but does not confirm that the entire enhancer is present on the transgene. Similarly, the partial knockdown of OR gene expression seen for single enhancer deletions does not clearly demarcate the functional extent of the enhancer sequence. In contrast, the deletion of TE1 and TE2 completely silences olfactory TAAR gene expression—clearly defining the functional boundaries of these enhancers.

Both T-elements and known OR enhancers comprise conserved HD binding sites, suggesting a common choice mechanism. However, previous studies have highlighted differences between TAAR and OR gene expression^40,41^. The TAARs appear to occupy a distinct nuclear compartment when compared to the ORs^41^. Moreover, ChIP-seq data assayed across the population of all mature OSNs show that the TAAR cluster lacks heterochromatic histone marks (H3K9Me3 and H4K20Me3) that are typically associated with class II OR clusters^33,40^. In addition, the available data indicate that the T-elements lack prominent binding of Lhx2, Ldb1, and Ebf1, which are features of some OR enhancers. One caveat to interpreting the ChIP-seq data is that TAAR-expressing OSNs represent a small fraction of the total number of mature OSNs. It is possible that repressive marks and transcription factor binding in the TAAR cluster might only be apparent in TAAR-expressing OSNs.

Despite these issues, the data indicate two things. First, in a majority of OSNs (most of which express class II ORs), the TAAR cluster is not marked in the same way as class II OR clusters. This suggests that the TAAR cluster may be silenced in OR-expressing OSNs independent of OR-typical heterochromatic marks. Second, in a majority of OSNs, the T-elements do not exhibit robust Lhx2 and Ldb1 binding to the same extent as many OR enhancers. This might suggest that the conserved HDs do not play a role in T-element function, or that the conserved HD sites bind factors other than Lhx2. Alternately, the T-element HD sites may be unable to associate with Lhx2 in most OSNs. One way to explain the ChIP-seq data is that the TAAR cluster may be inaccessible in OR expressing neurons—something that could be accomplished by repression of T-element function (see below).

Previous work has shown that subpopulations of OSNs (either across zones, or within a zone) are restricted to express subsets of OR genes^6,16,51–53^. The mechanisms underlying cell-type-specific OR choice restrictions are not understood. One way to create a choice restriction would be to induce (or inhibit) expression of OR genes by activating (or repressing) *cis*-acting enhancers that coordinate expression in OR clusters. Such a mechanism was recently proposed for the class I OR cluster^54^. Likewise, one way to include or exclude TAAR expression from a population of OSNs might be to simultaneously control the function of TE1 and TE2. An intriguing possibility is that the SHiTE motif, which is common to both elements, could mediate such coordinated enhancer control. SHiTE might serve as a cell-type-specific activator of enhancer function. However, given that both TE1 and TE2 contain conserved HD sites, which are known to promote OR gene choice, it seems feasible that SHiTE might mediate repression of a default propensity for expression. We note that SHiTE contains a sequence AAAGTTT which is the reverse complement of AAACTTT, which is conserved in the class I OR enhancer J^20^ and that is part of an extended HD motif^30^. While the contribution of the class I motif to gene choice is unclear^55^, there may be an as yet uncovered common function.

Our discovery of cooperative, *cis*-acting enhancers in the TAAR cluster that share common features with OR enhancers suggests that TAAR and OR gene choice proceed via a fundamentally similar mechanism. Thus, identifying which characteristics of gene choice are common between TAARs and ORs will help elucidate mechanisms that are essential for olfactory singular expression.

## Methods

All experimental procedures were approved by the Northwestern University Institutional Animal Care and Use Committee.

### Mouse strains

The T4-RFP (Taar4-IRES-tauCherry) and ΔT4-RFP (GAP-Cherry→Taar4) strains were described previously^6^.

#### ΔT4-YFP transgenes

A 129S7 BAC clone^56^ (bMQ-215O19) containing the Taar4 locus was modified via recombineering by the insertion of an AscI flanked galK cassette 3 nt downstream of the Taar4 coding sequence. A 7.9 kb fragment of the modified BAC (corresponding to GRCm39/mm39 chr10: 23,830,822-23,838,778) was isolated by gap repair, and the coding sequence replaced with that of Venus YFP^57^ preceded by a Kozak consensus (GCCACCATG). This base transgene (YFP → T4tg) contains ~2.3 kb upstream of the Taar4 transcription start site and 1.3 kb downstream of the coding sequence (including the endogenous polyadenylation site), and was flanked with PmeI sites for linearization. Enhancers (5x21HD, TE1 and TE2) were amplified by PCR and inserted into a XhoI site that is ~2 kb upstream of the transcriptional start. 5xHD-ΔT4YFPtg contains a 5 copy repeat of the extended 21 bp homeodomain sequence (ACATAACTTTTTAATGAGTCT), as previously described^30^. TE1-ΔT4YFPtg and TE2-ΔT4YFPtg contain one copy of TE1 (corresponding to GRCm39/mm39 chr10: 23,806,280-23,806,978) or TE2 (chr10: 23,863,682-23,864,027), respectively. Transgenes were linearized with PmeI purified, and injected into C57BL/6 J zygotes to generate transgenic founders.

#### ΔTE1, ΔTE2, ΔTE1/2 CRISPR alleles

PAM sequences on the 5′ and 3′ ends of each putative enhancer element were targeted with two guide RNAs each. The recognition sequences were cloned into pX458 (Addgene #48138) and the gRNAs transcribed in vitro using T7 RNA polymerase. The gRNAs and wild-type Cas9 RNA were injected into mouse zygotes that were compound heterozygous for two mutations: AU1-Taar1-IRES-tauVenus and Taar4-IRES-tauCherry. Founders were screened by PCR and direct sequencing. Mutant alleles with the following deletions were selected: GRCm39/mm39 chr10: 23,806,234-23,807,226 (993 bp) for ΔTE1, and chr10: 23,863,677-23,864,656 (980 bp) for ΔTE2, each isolated in *cis* with the AU1-Taar1-IRES-tauVenus mutation. An allele with deletions of both elements in tandem (ΔTE1/2) was also selected: chr10: 23,806,230-23,807,225 (996 bp) for ΔTE1 and chr10: 23,863,677-23,864,655 (979 bp) for ΔTE2, and was isolated in *cis* with the Taar4-IRES-tauCherry mutation. All of the alleles were backcrossed for six generations onto a C57BL/6 J background, then intercrossed. For all mutations, homozygous mice appear healthy and show normal fertility. Mice were housed in a specific-pathogen free barrier vivarium on a 14/10 light/dark cycle at 21–24 °C and 50% relative humidity with access to food and water ad libitum.

### Histology

Postnatal day 30 mice were anesthetized and transcardially perfused with 4% paraformaldehyde. The nose was dissected and post-fixed at 4 °C overnight, decalcified in 0.5 M EDTA overnight, and cryoprotected in 15% sucrose for 1 h and 30% sucrose overnight (both at 4 °C). OCT-embedded samples were cryosectioned at 12 μm. Combined in situ hybridization/immunohistochemistry in transgenic lines^16,58^ was performed using digoxigenin-labeled riboprobes and anti-digoxigenin-alkaline phosphatase Fab fragments at 1:1000 dilution (Roche, 11093274910). Proteinase K (10 μg/ml; Takara 740396) treatment time was adjusted to allow for immunostaining. YFP protein was detected with a chicken anti-GFP antibody (Abcam, ab13970) at 1:500, coupled with a rabbit anti-chicken peroxidase secondary (Invitrogen, 61-3120) 1:500. Tyramide signal amplification was performed using TSA amplification reagent (Akoya Biosciences) at 1/10 of the prescribed biotinyl-tyramide concentration^58^. Probes for ORs/TAARs were transcribed using T7 or SP6 RNA polymerase from cloned and sequenced partial or full-length coding sequence templates that were amplified from olfactory epithelium cDNA or genomic DNA. Template plasmids were linearized and purified by phenol:chloroform extraction. The probe pools were— class I: *olfr623*, *olfr578*, *olfr691*, *olfr672*, *olfr668*, and *olfr545*; class II: *olfr749*, *olfr745*, *olfr771*, *olfr1031*, *olfr62*, and *olfr247*; TAAR: *taar2*, *taar4*, *taar5*, *taar6*, *taar7a*, and *taar9*.

All fluorescence images were acquired using a Zeiss LSM880 laser scanning confocal microscope using Zen 2.3 SP1 software (Carl Zeiss Microscopy). For whole-mount analysis of olfactory bulbs and epithelia, genetically encoded fluorescent markers were visualized in unfixed samples. Wholemount images were collected as tile-scanned z-stacks and displayed as flattened projections.

### Gene expression analysis

#### qPCR

Olfactory mucosa was isolated from P30 TAAR element deletion mice and matching wild-type littermate controls. RNA was purified using the Qiagen RNeasy Kit (#74134) with on-column DNase treatment using Qiagen RNase-free DNase (#EN0521). Oligo-dT primed cDNA was synthesized using SuperScript III Reverse transcriptase (#18080044). qPCR was performed according to MiQE guidelines^59^, using iQ SYBR Green (#1708884). ΔCt values were calculated using the geometric mean of the housekeeping genes *Bgus*, *Gnal*, and *Ncam*. Primers were tested for efficiency (95–105%). Primer sequences are found in Supplementary Table 1.

#### RNAseq

Olfactory mucosa was isolated from postnatal day (P30) TAAR element deletion mice and matching wild-type littermate controls. RNA was purified using the Qiagen RNeasy Kit (#74134) and on-column DNase treatment was performed using Qiagen RNase-free DNase (#EN0521). Stranded, OligodT selected cDNA libraries were synthesized, multiplexed, and run on an Illumina HiSeq4000 (PE100) by the University of Chicago Genomics Facility. Reads were aligned to a modified GRCm38.p6 mouse genome containing extended TAAR exon and UTR information using STAR aligner v2.4.2^60^. Differential expression analysis was performed using DeSeq2 v1.30.1^61^ using default settings.

### Statistics and reproducibility

qPCR data were analyzed by one-way ANOVA corrected for multiple comparisons (Dunn–Šidák) on the raw delta Ct values to test the null hypothesis of equal Ct values between samples. Differential expression in RNAseq data was estimated using the Wald test correcting for multiple comparisons using the method of Benjamini and Hochberg. All tests were done using Prism v7 (GraphPad) and R v1.2 (www.r-project.org).

Gene expression patterns in transgenic mice were documented in at least 3–6 animals for each independent line. RNAseq and in situ hybridization experiments were performed once using the number of independent samples (animals) indicated in the main text.

### Reporting summary

Further information on research design is available in the Nature Research Reporting Summary linked to this article.

## Supplementary information

Supplementary Information

Reporting Summary

## Data Availability

RNAseq datasets generated and analyzed during the currents study are available via the Geo database (accession #GSE171241). Previously published datasets^21,27^ that were analyzed during the current study are also available (accession #GSE93570 for ATAC-seq, Lhx2 ChIP-seq, Ebf ChIP-seq and GSE112152 for Ldb1 ChIP-seq). Eutherian conservation track analyzed in the current study can be found on the UCSC Genome Browser. Source data are provided with this paper.

## Source data

Source Data
