# Supplementary material for: Olfactory expression of trace amine-associated receptors requires cooperative cis-acting enhancers
Source: Nat Commun. 2021 Jun 18;12:3797. doi: 10.1038/s41467-021-23824-3 (PMC8213819; doi:10.1038/s41467-021-23824-3)
Supplement: Supplementary file 1 — Supplementary Information [file 41467_2021_23824_MOESM1_ESM.pdf]

Supplementary Data

Olfactory expression of trace amine-associated receptors requires cooperative *cis*-acting enhancers.

Shah et al.

Supplementary Table 1: Sequences for primers used in qPCR analysis

Primer	Sequence (5' – 3')
TAAR2 fwd	CGGATTCACCATCATGCCAT
TAAR2 rev	CTAAGCATCAGGTCGAAGCT
TAAR3 fwd	GTCATGGGGGTGTTTCTAG
TAAR3 rev	AGAAGATCCAGCACTATGATG
TAAR4 fwd	TGGAGCTCCCCAGAAGTAC
TAAR4 rev	GCTGCTTGAAGTGGGCAATAG
TAAR5 fwd	CTTCTTAACTTCATCACCCCAC
TAAR5 rev	AATCAACAGTCGGTGTCCTTG
TAAR6 fwd	CAACTCAGCCATGAACCCTC
TAAR6 rev	CACCTGGCCACTCATAATAAC
TAAR7a fwd	TGATCAATCTAGAACCATGAATG
TAAR7a rev	GACCTGTTCAGGTTCTCATAG
TAAR7b fwd	ATTTCAGGCAGCTACACTCC
TAAR7b rev	TCAGCACAGGCCAAGGACA
TAAR7d fwd	TTCCATTCTTGCTTTGAAGGG
TAAR7d rev	AGCACAAGTGGAAGAGAGAG
TAAR7e fwd	AAGTGATGAATTATGCTTCTGCC
TAAR7e rev	AATCCTCAGATTTCACCAAAATG
TAAR7f fwd	CTGGGTCTTTATCAACTTCTC
TAAR7f rev	ATCATCACAAGGGTGGGAATG
TAAR8a-c fwd	GTGAATCATCCTCAGAGAGTC
TAAR8a-c rev	TATGGGAGCCACGAGACC
TAAR9 fwd	CCTCGAGCCATCCTCTATG
TAAR9 rev	CAGGAAGTTCGTAGGCGTG
Olfr160 fwd	GAGGGCTAACTAACAGGCCA
Olfr160 rev	TCAAGGTGATCATGCCCAGA
Olfr16 fwd	TTGTTGGTTTGTGGGTCTTTAG
Olfr16 rev	CAAAAGGGCAAATGGAACATG
Olfr545 fwd	ATTGTCTACCGTGTGGCTC
Olfr545 rev	AGGAATGACCTTACCAGGTG
Olfr73 fwd	CCGCAACCCTCTACTCTAC
Olfr73 rev	ATTCAAGGCAGAGCAGGTC
Olfr961 fwd	ACTCAATAGTGCCATCATCTTG
Olfr961 rev	TGGTTTCCTTCACAGTCTCA
NCAM fwd	ACCATCTACAACGCCAACATC
NCAM rev	CATCACAGACAATCACAGCATC
Gnal fwd	CAACCCCGAGGAAAAGAAG
Gnal rev	CAGTGGAACTGGAGGTATG
Bgus fwd	GCTGATCACCCACACCAAAG
Bgus rev	CACAGATAACATCCACGTACG

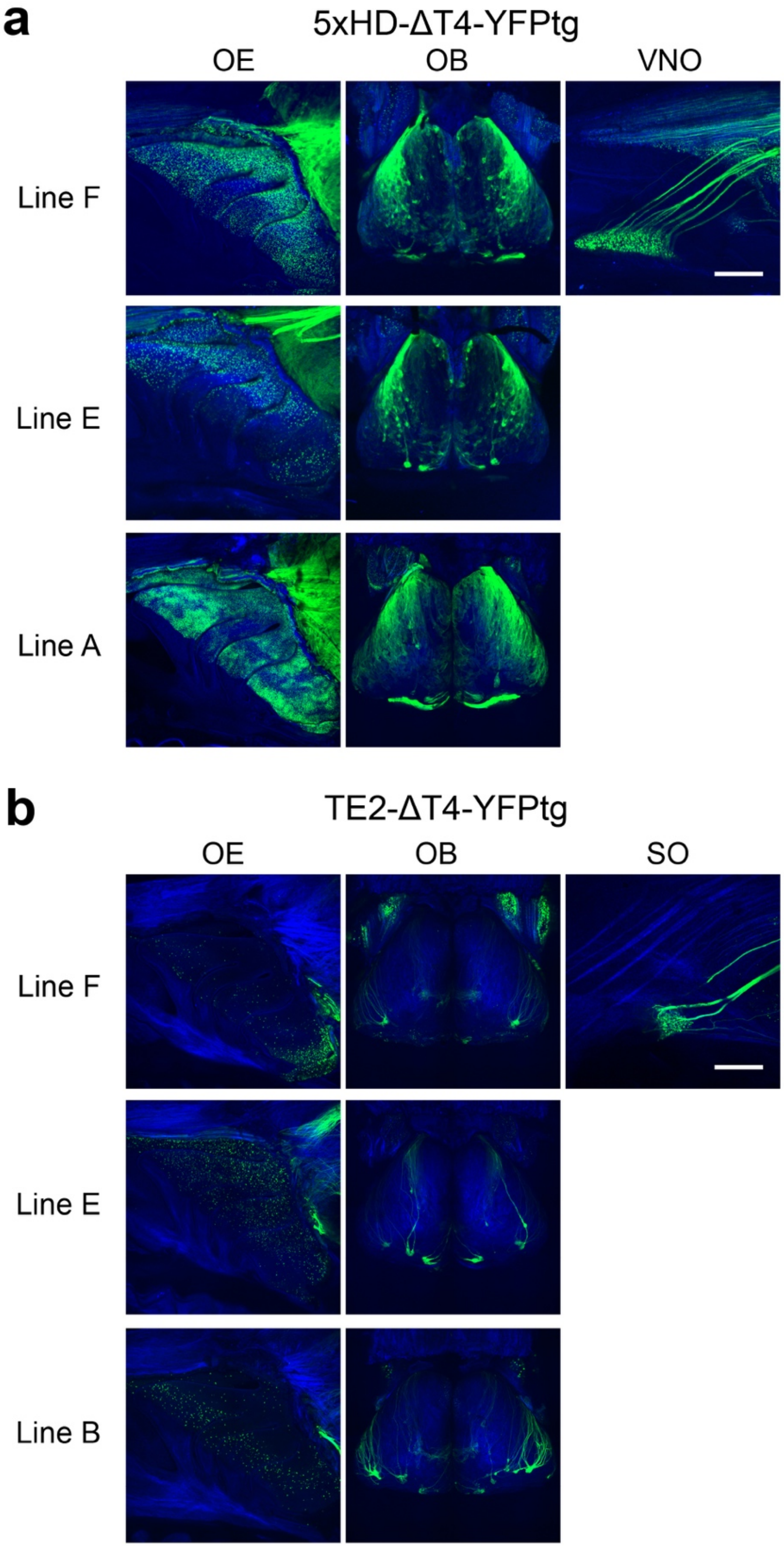

Figure S1. Characterization of alternate transgenic lines
Confocal images of YFP expression (green) in independent transgenic lines not depicted in main figures. (a) wholemounts of olfactory epithelium (OE), olfactory bulb (OB) and vomeronasal organ (VNO) for 3 out of 5 5xHD-ΔT4-YFPtg lines. (b) wholemounts of OE, OB and septal organ (SO) for 3 out of 5 TE2-ΔT4-YFPtg lines. Scale bars are 1000 µm in OE, OB and VNO panels, and 500 µm in SO panel.

a

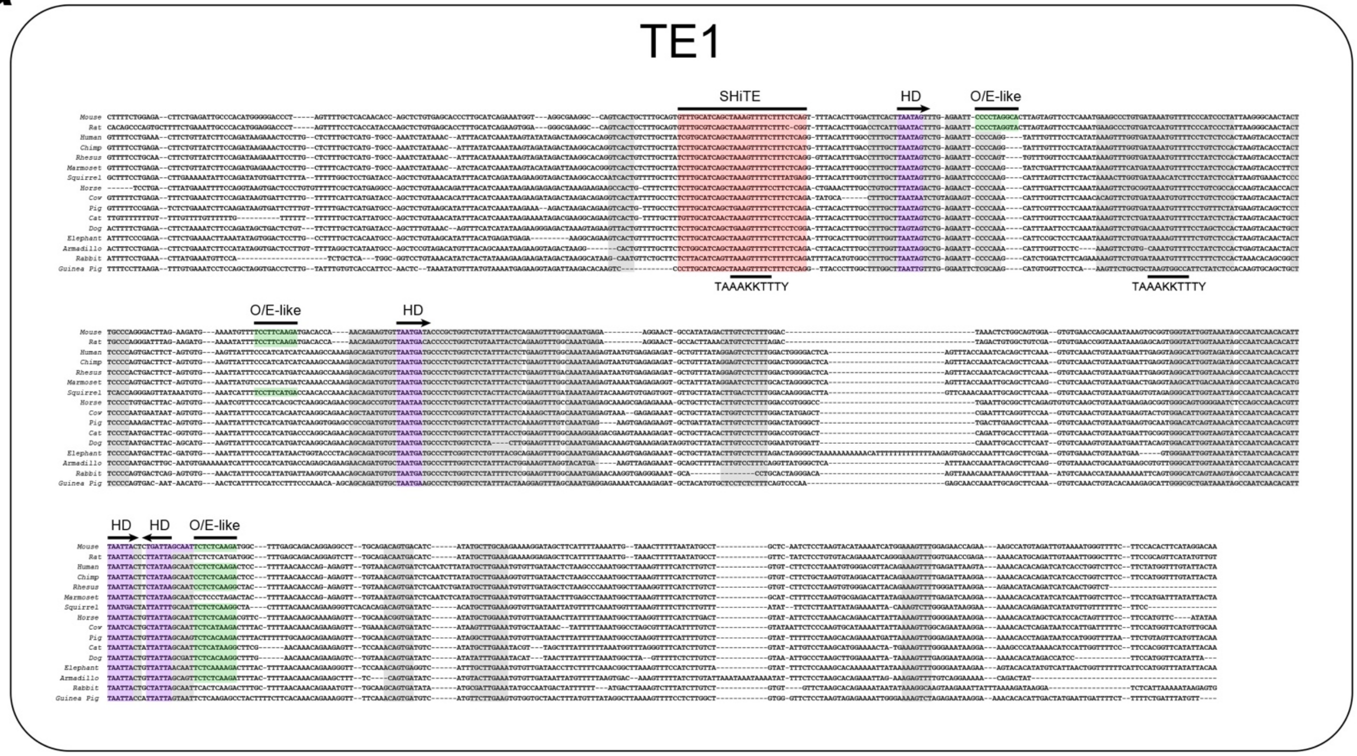

b

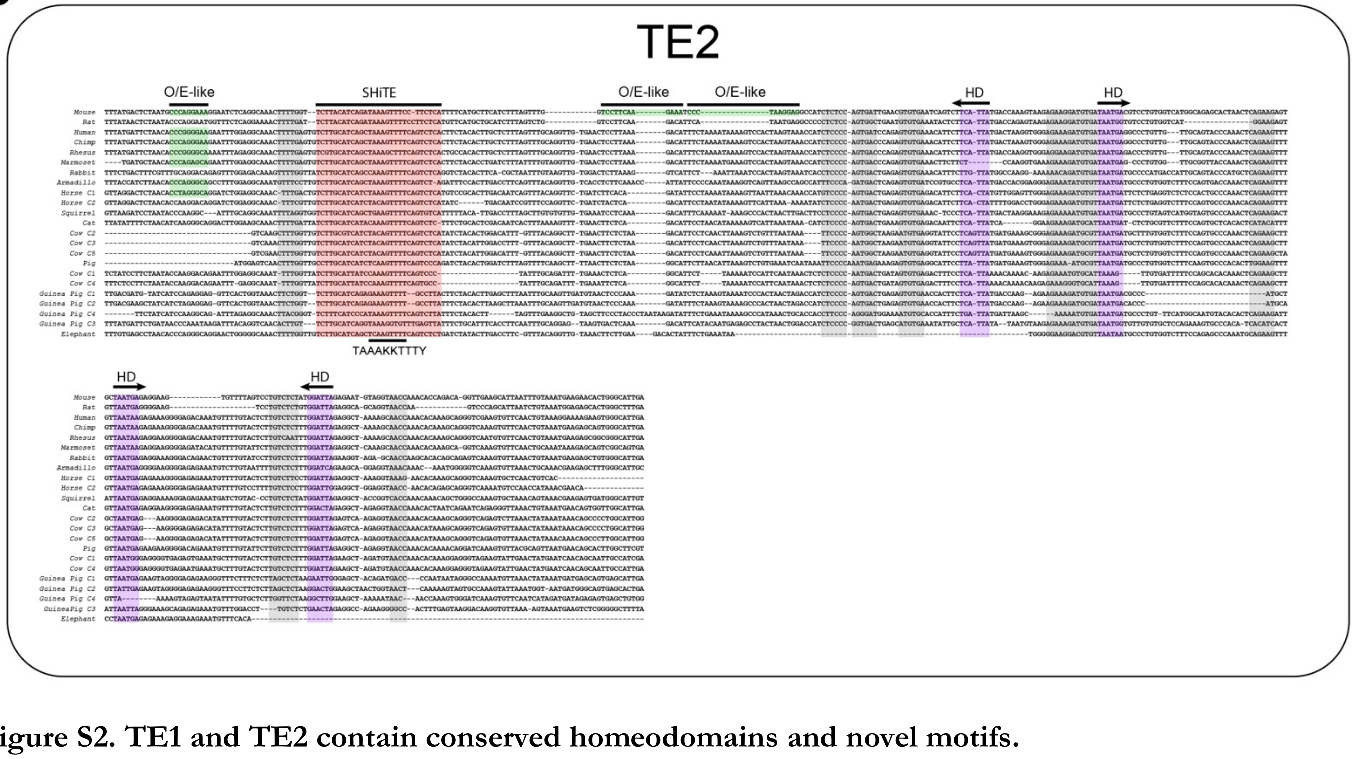

Figure S2. TE1 and TE2 contain conserved homeodomains and novel motifs.
Alignment of the full TE1 (a) and TE2 (b) sequences across 16 eutherian mammals. Some animals contain multiple homologous copies of TE2. Conserved HD sites are shaded in purple. The novel Shared Homology motif (SHiTE) is shaded in red. Other conserved blocks are shaded in grey. Putative O/E-like sites are highlighted in green. The sequence TAAAKKTTTY that is found twice in TE1 and once in TE2 is indicated.

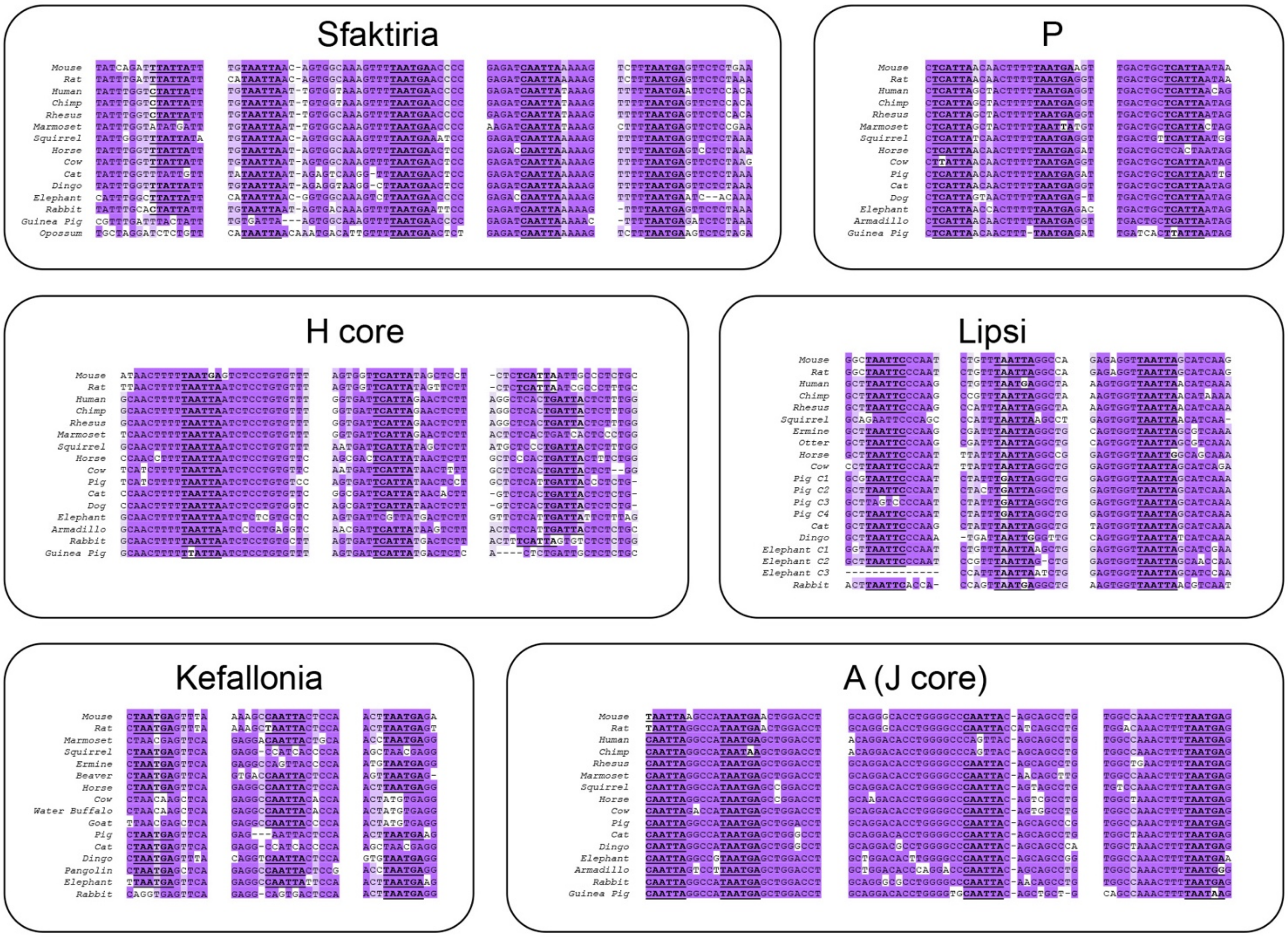

Figure S3. Conserved homeodomain sites in known functional OR enhancers.
Sequence alignments showing blocks of conservation that encompass conserved HD binding sites in 6 olfactory enhancers across Eutherian mammals. HD sites (TAATNN) are shown in bold underline. Conserved sequences are shaded in purple.

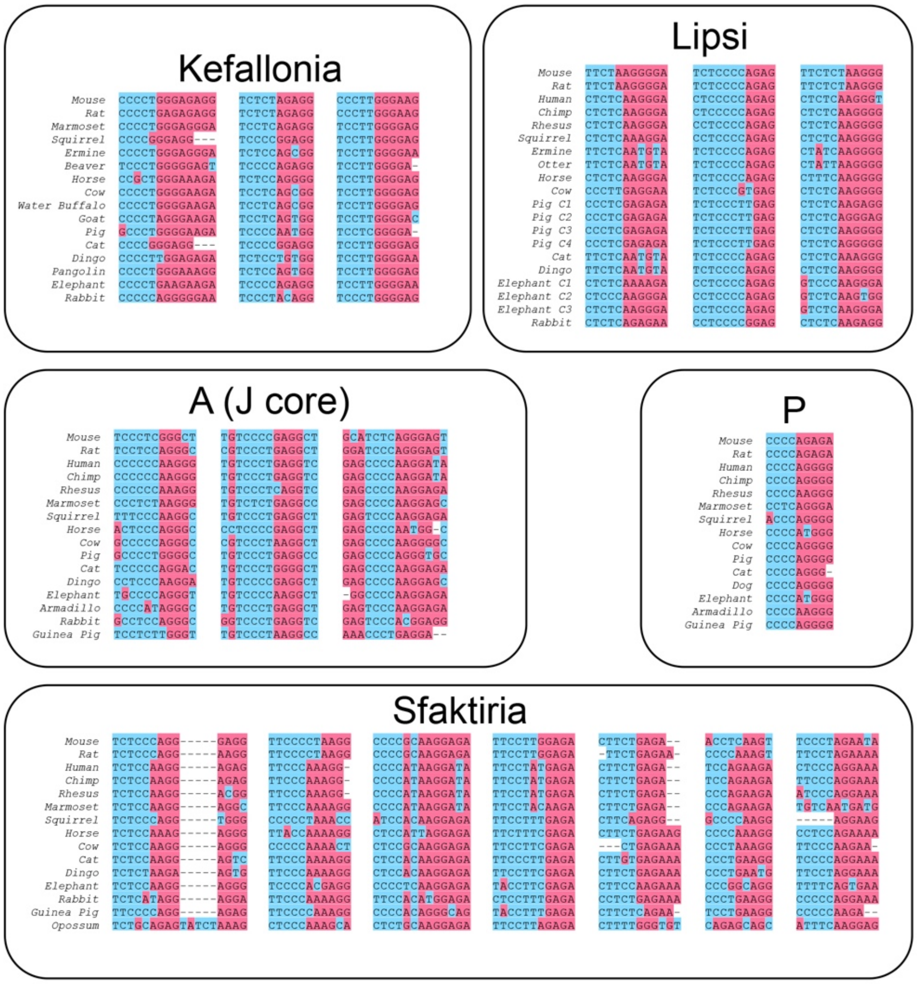

Figure S4. Conserved O/E-like sites in known functional OR enhancers.
Sequence alignments showing conserved O/E-like sites (approximate matches to the consensus YCCCNNGGGR) in olfactory enhancers across a subset of eutherian mammals. Sequence is highlighted purines (magenta) and pyrimidines (cyan). The enhancer H is not included as it lacks a conserved O/E-like site.

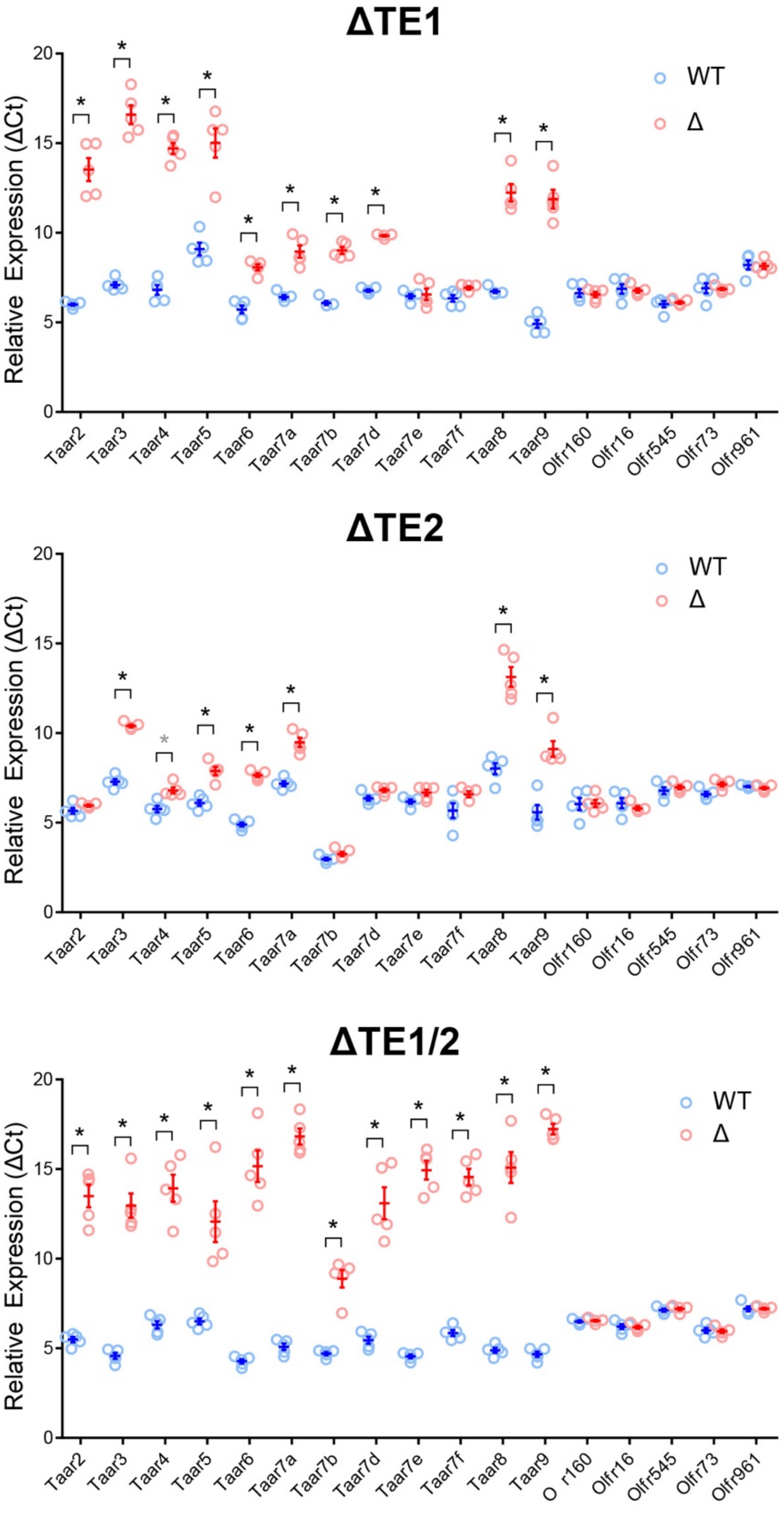

Figure S5. Raw qPCR data for statistical analysis

Raw ΔCt data for ΔTE1, ΔTE2 and ΔTE1/2 mice shown in Fig. 6 and 8 (n=5 animals per genotype). Wild-type shown in blue and deletion in red (error bars indicate mean±SEM). Larger ΔCt values indicate fewer transcript copies (lower expression). Pairwise comparisons from one-way ANOVA are indicated. Black * = $p<0.01$; grey * = $p<0.05$ two-sided, one-way ANOVA, Dunn–Šidák correction for multiple comparisons.
