# Supplementary material for: Olfactory expression of trace amine-associated receptors requires cooperative cis-acting enhancers
Source: Nat Commun. 2021 Jun 18;12:3797. doi: 10.1038/s41467-021-23824-3 (PMC8213819; doi:10.1038/s41467-021-23824-3)
Supplement: Supplementary file 2 — Reporting Summary [file 41467_2021_23824_MOESM2_ESM.pdf]

Corresponding author(s): Bozza, Thomas

Last updated by author(s): Apr 5, 2021

Reporting Summary

Nature Research wishes to improve the reproducibility of the work that we publish. This form provides structure for consistency and transparency in reporting. For further information on Nature Research policies, see our Editorial Policies and the Editorial Policy Checklist.

Statistics

For all statistical analyses, confirm that the following items are present in the figure legend, table legend, main text, or Methods section.

n/a	Confirmed	
☐	☒	The exact sample size (n) for each experimental group/condition, given as a discrete number and unit of measurement
☐	☒	A statement on whether measurements were taken from distinct samples or whether the same sample was measured repeatedly
☐	☒	The statistical test(s) used AND whether they are one- or two-sided<br>*Only common tests should be described solely by name; describe more complex techniques in the Methods section.*
☒	☐	A description of all covariates tested
☒	☐	A description of any assumptions or corrections, such as tests of normality and adjustment for multiple comparisons
☒	☐	A full description of the statistical parameters including central tendency (e.g. means) or other basic estimates (e.g. regression coefficient) AND variation (e.g. standard deviation) or associated estimates of uncertainty (e.g. confidence intervals)
☐	☒	For null hypothesis testing, the test statistic (e.g. F, t, r) with confidence intervals, effect sizes, degrees of freedom and P value noted<br>*Give P values as exact values whenever suitable.*
☒	☐	For Bayesian analysis, information on the choice of priors and Markov chain Monte Carlo settings
☒	☐	For hierarchical and complex designs, identification of the appropriate level for tests and full reporting of outcomes
☒	☐	Estimates of effect sizes (e.g. Cohen's d, Pearson's r), indicating how they were calculated

Our web collection on statistics for biologists contains articles on many of the points above.

Software and code

Policy information about availability of computer code

Data collection	Zeiss Zen 2.3 SP1
Data analysis	STAR Aligner v2.4.2, DESeq v 1.30.1, R v1.2, Prism v7

For manuscripts utilizing custom algorithms or software that are central to the research but not yet described in published literature, software must be made available to editors and reviewers. We strongly encourage code deposition in a community repository (e.g. GitHub). See the Nature Research guidelines for submitting code & software for further information.

Data

Policy information about availability of data

All manuscripts must include a data availability statement. This statement should provide the following information, where applicable:

- Accession codes, unique identifiers, or web links for publicly available datasets
- A list of figures that have associated raw data
- A description of any restrictions on data availability

RNAseq datasets generated and analyzed during the currents study are available via the Geo database (accession #GSE171241 [https://www.ncbi.nlm.nih.gov/geo/query/acc.cgi?acc=GSE171241]). Previously published datasets21, 27 that were analyzed during the current study are also available (accession #GSE93570 [https://www.ncbi.nlm.nih.gov/geo/query/acc.cgi?acc=GSE93570] for ATAC-seq, Lhx2 ChIP-seq, Ebf ChIP-seq and GSE112152 [https://www.ncbi.nlm.nih.gov/geo/query/acc.cgi?acc=GSE112153] for Ldb1 ChIP-seq). Eutherian conservation track analyzed in the current study can be found on the UCSC Genome Browser [https://genome.ucsc.edu/cgi-bin/hgTracks?db=mm10&lastVirtModeType=default&lastVirtModeExtraState=&virtModeType=default&virtMode=0&nonVirtPosition=&position=chr10%3A23909843%2D24113677&hgsid=1072811905_YqWTNkkw5o2DBczAwQQ2ypqAjkDX]. Source data are provided with the paper as Source Data File.

Field-specific reporting

Please select the one below that is the best fit for your research. If you are not sure, read the appropriate sections before making your selection.

- [x] Life sciences
- [ ] Behavioural & social sciences
- [ ] Ecological, evolutionary & environmental sciences

For a reference copy of the document with all sections, see nature.com/documents/nr-reporting-summary-flat.pdf

Life sciences study design

All studies must disclose on these points even when the disclosure is negative.

Sample size	Sample sizes for histology and in situ hybridization were determined based on previous data on variability of OR gene expression in mice (Bozza et al. Neuron 2009 61(2):220-33). Sample sizes for qPCR and RNAseq were set at 5 and 4 mice respectively based on variability in gene OR expression observed in pilot studies, though no formal power analysis was performed.
Data exclusions	No data were excluded from the analysis.
Replication	Gene expression patterns in transgenic mice were documented in at least 3-6 animals for each independent line. RNAseq and in situ hybridization experiments were performed once using the indicated number of independent animals.
Randomization	No randomization was necessary. Animals were selected based on genotype. All experimental groups used littermate controls.
Blinding	Experimental data for cell counts were collected with the observed blinded to experimental group or probe set. For RNAseq and qPCR gene expression analysis, no blinding was necessary as the data collection methods were automated and consistent across samples and groups.

Reporting for specific materials, systems and methods

We require information from authors about some types of materials, experimental systems and methods used in many studies. Here, indicate whether each material, system or method listed is relevant to your study. If you are not sure if a list item applies to your research, read the appropriate section before selecting a response.

Materials & experimental systems

n/a	Involved in the study	
[ ]	[x]	Antibodies
[x]	[ ]	Eukaryotic cell lines
[x]	[ ]	Palaeontology and archaeology
[ ]	[x]	Animals and other organisms
[x]	[ ]	Human research participants
[x]	[ ]	Clinical data
[x]	[ ]	Dual use research of concern

Methods

n/a	Involved in the study	
[x]	[ ]	ChIP-seq
[x]	[ ]	Flow cytometry
[x]	[ ]	MRI-based neuroimaging

Antibodies

Antibodies used	Chicken Anti-GFP (Abcam, ab13970)<br>Rabbit anti-chicken peroxidase (Invitrogen, 61-3120)<br>Anti-Digoxigenin-AP, Fab fragments (Roche, 11093274910)
Validation	Anti-Digoxigenin-AP, Fab fragments (Roche, 11093274910) were documented in Ishii et al., Journal of Neurocytology volume 33, 657–669 (2004).<br>Chicken Anti-GFP (Abcam, ab13970) is documented https://www.abcam.com/gfp-antibody-ab13970.html<br>Rabbit anti-chicken peroxidase (Invitrogen, 61-3120) is documented in manuscripts listed https://www.citeab.com/antibodies/2500530-61-3120-rabbit-anti-chicken-turkey-igy-secondary-a

Animals and other organisms

Policy information about studies involving animals; ARRIVE guidelines recommended for reporting animal research

Laboratory animals	Mice (Mus musculus) were maintained in a specific pathogen free vivarium in a 14hr/10hr light/dark cycle kept between 21-24 degrees C at ~50% relative humidity. Food and water were available ad libitum. Mice of both sexes were used and sex was analyzed as a factor. When not significant, data from both sexes were pooled. Wholemount analysis was done in mice at P20-22. For all other analyses, mice were used at P30.

Wild animals	None
Field-collected samples	None
Ethics oversight	Northwestern University IACUC

Note that full information on the approval of the study protocol must also be provided in the manuscript.
